# Severity of Retinopathy Parallels the Degree of Parasite Sequestration in the Eyes and Brains of Malawian Children With Fatal Cerebral Malaria

**DOI:** 10.1093/infdis/jiu592

**Published:** 2014-10-28

**Authors:** Valentina Barrera, Paul Stephenson Hiscott, Alister Gordon Craig, Valerie Ann White, Danny Arnold Milner, Nicholas Alexander Venton Beare, Ian James Callum MacCormick, Steve Kamiza, Terrie Ellen Taylor, Malcolm Edward Molyneux, Simon Peter Harding

**Affiliations:** 1 Department of Eye and Vision Science, Institute of Ageing and Chronic Disease, University of Liverpool; 2 Liverpool School of Tropical Medicine; 3 St. Paul's Eye Unit, Royal Liverpool University Hospital, United Kingdom; 4 Department of Pathology and Laboratory Medicine; 5 Department of Ophthalmology and Visual Science, University of British Columbia and Vancouver General Hospital, Canada; 6 Anatomic and Clinical Pathology, Brigham and Women's Hospital; 7 Immunology and Infectious Diseases, Harvard School of Public Health, Boston, Massachusetts; 8 Department of Osteopathic Medical Specialties, College of Osteopathic Medicine, Michigan State University, East Lansing; 9 Malawi-Liverpool-Wellcome Trust Clinical Research Programme; 10 Department of Histopathology; 11 Blantyre Malaria Project, College of Medicine, University of Malawi, Blantyre

**Keywords:** malarial retinopathy, cerebral malaria, *Plasmodium falciparum* malaria, pediatric coma, clinicopathological correlation, neurovasculature, parasite sequestration, microvascular congestion, vascular pathology, histopathology

## Abstract

***Background.*** Malarial retinopathy (MR) has diagnostic and prognostic value in children with *Plasmodium falciparum* cerebral malaria (CM). A clinicopathological correlation between observed retinal changes during life and the degree of sequestration of parasitized red blood cells was investigated in ocular and cerebral vessels at autopsy.

***Methods.*** In 18 Malawian children who died from clinically defined CM, we studied the intensity of sequestration and the maturity of sequestered parasites in the retina, in nonretinal ocular tissues, and in the brain.

***Results.*** Five children with clinically defined CM during life had other causes of death identified at autopsy, no MR, and scanty intracerebral sequestration. Thirteen children had MR and died from CM. MR severity correlated with percentage of microvessels parasitized in the retina, brain, and nonretinal tissues with some neuroectodermal components (all *P* < .01). In moderate/severe MR cases (n = 8), vascular congestion was more intense (ρ = 0.841; *P* < .001), sequestered parasites were more mature, and the quantity of extraerythrocytic hemozoin was higher, compared with mild MR cases (n = 5).

***Conclusions.*** These data provide a histopathological basis for the known correlation between degrees of retinopathy and cerebral dysfunction in CM. In addition to being a valuable tool for clinical diagnosis, retinal observations give important information about neurovascular pathophysiology in pediatric CM.

Cerebral malaria (CM) is a severe complication of infection with *Plasmodium falciparum* that has a disproportionate impact on children in sub-Saharan Africa. CM is defined clinically as peripheral parasitemia with coma not directly attributable to convulsions, hypoglycemia, concomitant infections, or any other identifiable cause [1, 2]. This definition is broad, and where malaria is endemic it may misclassify CM in up to 25% of cases, when postmortem histopathological analysis of the brain is the reference standard [2].

Pediatric CM is associated with several retinal signs collectively known as malarial retinopathy (MR) [3–5]. These include retinal whitening, vessel discoloration, retinal hemorrhages, and papilledema [6]. MR is an important finding in children with suspected CM because it is the only clinical sign recognized during life that distinguishes between histopathologically confirmed CM and cases that meet the broad clinical definition of CM but actually have another cause of death [2]. The severity of retinopathy in CM is associated with death [7], and the number of hemorrhages in the retina before death is correlated with the density of hemorrhages in the brain at autopsy [8]. Since the retina and brain share many features, including their neuroectodermal origin, these empirical associations suggest that retinal observations in CM may approximate similar but unseen cerebral pathology [4]. However, a direct quantitative comparison of the degree of sequestration in retina and brain has not been carried out.

*P. falciparum* grows in human red blood cells (RBCs) over a 48-hour cycle [9]. At 18–20 hours after invasion, the parasite matures and parasitized RBCs (pRBCs) sequester in the microvasculature of various tissues, including neural tissue. In CM, intracerebral accumulation of sequestered pRBCs is linked to vascular pathology [10], with changes potentially causing moderate blood brain barrier dysfunction [11]. The pigment hemozoin is created by the consumption of hemoglobin by parasites [12], and because it becomes unambiguously visible microscopically at 30–34 hours [13], it is a useful indicator of the stage of parasite development. Hemozoin becomes extraerythrocytic when is released into the vessel lumen after schizont rupture [14–18].

Vascular congestion is a pathological process occurring in vessels because of increased accumulation of blood cells and impaired outflow from the tissue, and it has recently been identified as a potential mechanism of coma during CM. Intense sequestration of pRBCs, enhanced by adherence of noninfected RBCs to pRBCs (known as rosetting), may lead to vascular congestion in the brain, which in adults has been associated with deeper levels of premortem coma and a shorter time to death [19].

To further elucidate the relationship between and the parasitological basis for MR and CM, we conducted a clinicopathological study of intact retinas from eyes obtained as part of a previous long-term autopsy study of Malawian children who died with coma and *P. falciparum* parasitemia. We postulated that the presence of sequestration in retinal vessels would distinguish between CM and non-CM coma and that it would parallel neurovascular sequestration in the brain. Moreover, we hypothesized that there may be an association between the severity of retinopathy diagnosed during life and the degree of parasite sequestration, the latter including levels (percentage of parasitized vessels), intensity (number of pRBCs sequestered), and maturation stage of sequestered pRBCs. In addition to neural tissue, the eye contains tissues derived from nonneuroectodermal sources, as well as tissues with some neuroectodermal components, in addition to those derived from the mesoderm and ectoderm (Supplementary Materials). We hypothesized that the density of sequestration would be greatest in tissues that have the same embryological origin as the brain and least in tissues fully derived from nonneural progenitors. If correct, this finding would support the concept that neuroectodermal origin is a factor in the pathogenesis of pediatric CM and that clinically observed signs of malarial retinopathy are likely to reflect cerebral pathology, because the retina and brain share a common embryological heritage [4, 20].

## MATERIALS AND METHODS

### Study Subjects and Clinical Eye Examination

Individuals in the autopsy study [2] were recruited from among children admitted to the Queen Elizabeth Central Hospital in Blantyre, Malawi, between 1996 and 2010 with a clinical diagnosis of CM and whose parent/guardian gave informed consent. The definition of CM was presence of coma (Blantyre coma score ≤2 [21]) and *P. falciparum* parasitemia in the absence of any other identifiable cause of coma (including meningitis, hypoglycemia, or postictal state of ≤2 hours).

Subjects for our study of MR were patients from the archive of this autopsy study who had had a full clinical eye examination during life. Based on our previously published findings of the prognostic significance of severity of MR [7], cases were available from 3 groups: no MR, mild MR, and moderate/severe MR. pRBC counts in the brain, determined by histological analysis, were available for the same patients, for whom parasite sequestration in cerebral capillaries was quantified [16] (a detailed description of CM classification and cerebral pathology is available in Supplementary Table 1). Clinical eye examinations were performed by indirect ophthalmoscopy before death. MR was graded according to a previously published system on standardized charts [6] with records made of retinal whitening (central and peripheral), retinal vessel discoloration, retinal hemorrhages, and papilledema. Mild MR was defined as the presence of the mildest positive category for any retinal sign(s) during clinical examination (vessel changes ≤2 quadrants, hemorrhages ≤5, composite central retinal whitening ≤2, and peripheral retinal whitening ≤1). Moderate/severe MR was defined as MR in which ≥1 retinal sign exceeded the mild category [7]. The presence of papilledema was also scored because it is the highest predictor of death in patients with MR [7]. If a patient died, autopsy was performed to international standards and eyes were removed. The last measurement of parasitemia was performed within 6 hours of death, and the patient's human immunodeficiency virus type 1 serological status was determined as previously described [2].

### Tissue Processing and Pathologic Microscopy

The eye specimens were coded and fixed in 10% v/v neutral buffered formalin and then processed and embedded in paraffin wax as previously described [22]. Tissue sections 3–4 µm thick were cut and stained with standard hematoxylin-eosin or Giemsa stains. All observations were performed by individuals who were masked to the patient's disease status. Eye histopathological analysis was conducted to quantify sequestration in microvessels (arterioles, venules, and capillaries) in the neural retina, optic nerve, choroid, ciliary body, iris, episclera/Tenon capsule, and extraocular muscles. Three different malaria parasite elements were scored after death in the microvasculature: unpigmented pRBCs (without hemozoin; known as immature forms), pigmented pRBCs (with intraerythrocytic hemozoin; known as mature forms), and extraerythrocytic hemozoin. These evaluations were made per vessel cross-section, across a minimum of 5 tissue sections stepped through a depth of at least 200 µm of tissue thickness for each case, in a standardized fashion as described elsewhere (Supplementary Materials) [2, 16, 22]. Parasite counts were reported for at least 100 vessels for each vessel subtype examined in each specimen for each ocular tissue. Both levels (expressed as a percentage of parasitized vessels) and intensity (expressed as the number of pRBCs sequestered) were reported for parasite sequestration. Microvascular congestion was measured using a method modified from that described by Ponsford et al [19] by summing the number of pRBCs and nonparasitized RBCs (npRBCs) in vessel subtypes (diameter range, 8–50 µm). Counted cells were recorded individually, and data were normalized and reported as pRBCs plus npRBCs per 100 parasitized microvessels.

### Statistical Analysis

After the quantitative evaluations were completed, specimen codes were broken, and the results were compared to the clinical data, using SPSS Statistics 20 (Supplementary Materials). Means ( ± SDs) are reported. The Student *t* test was used to compare parametric data between 2 groups. One-way analysis of variance (with the Bonferroni post-hoc modification) and the Kruskal–Wallis test were used to compare data across the 3 MR classification groups, for parametric and nonparametric data, respectively. Values of *P* ≤ .05 were considered statistically significant. Spearman bivariate analysis (with statistical significance defined as a *P* value of < .01) was used to correlate the percentage of vessels parasitized or the number of RBCs (npRBCs and pRBCs) with the MR ranking score, based on the sum of scores assigned to grading of vessel changes, hemorrhages, central and peripheral whitening, and papilledema (papilledema, if present, was weighted at a score of 2 to reflect the high odds of death associated with this factor [7]). To further investigate congestion in parasitized vessels, linear regression analysis was performed.

### Ethics Statement

The study was approved by the research ethics committees at the University of Malawi College of Medicine, Michigan State University, and the Liverpool School of Tropical Medicine and was performed in accordance with the Declaration of Helsinki. Written consent for the clinical eye examination was sought in English or in the language of the parent/guardian who gave the permission on the patient's behalf. If a patient died, additional informed written consent for autopsy was sought from the parent/guardian (as approved by the research ethics committees at the University of Malawi).

## RESULTS

**Figure 1.**
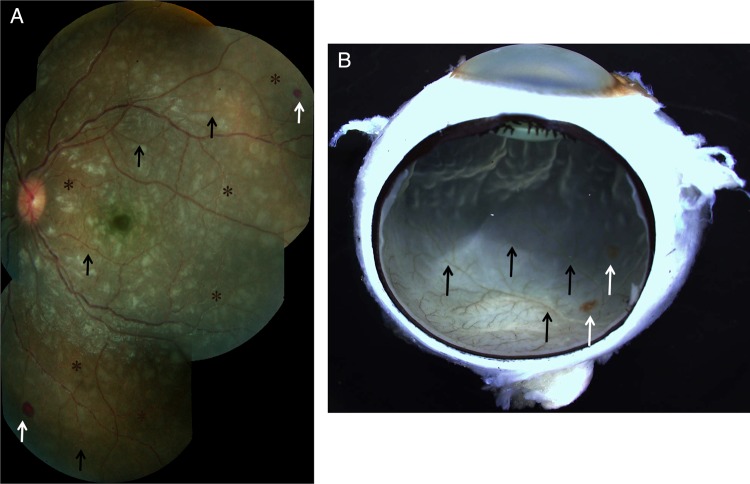
Clinical and gross pathology photographs for patient 3, who had moderate/severe MR. *A*, Color fundus montage image from the left eye. Retinal whitening, appearing as patches of opacification, can be seen in all retinal quadrants. Vessel discoloration, characterized by orange color changes, was seen (black arrows), frequently in association with vessel tortuosity (asterisks). Two hemorrhages are present at the retinal periphery (white arrows). *B*, Gross photograph, taken after death, of the enucleated eye of patient 3, showing retinal vessel discoloration and hemorrhages (black and white arrows, respectively).

A total of 18 patients from the archive met the clinical and consent criteria for inclusion in the study. Of these 18 patients, 5 had no MR, 5 had mild MR, and 8 had moderate/severe MR. Full details of the retinal clinical examinations are presented in Table 1, and additional clinical information is presented in Supplementary Table 1. The 5 children in the no MR group were found to have died from a nonmalaria cause (pneumonia or hepatitis) and had a low proportion of parasitized capillaries in the brain (Supplementary Table 1) [2, 16]. Although these children had peripheral *P. falciparum* parasitemia on admission, none of them had MR. In the 13 MR cases, no other causes of death were identified at autopsy, and in each case the amount of intracerebral sequestration of pRBCs exceeded 21% of capillaries, consistent with a diagnosis of CM (Supplementary Table 1) [2, 16]. The clinical signs of MR are shown in Figure 1 and Supplementary Figure 1.

**Table 1.** Master Table of Retinal Pathological Features and Scores for Each Subject

Patient	Eye	Vessel Changes		Retinal Hemorrhages		Retinal Whitening				Papilledema		Overall Score
		Quadrants Affected, No.	Score^a^	No.	Score^b^	Macular, DA	Foveal, FA	Central Retinal, Score^c^	Peripheral, Score	Present	Score^d^	
1	Right	4	3	≥51	4	1/3–1	1/3–2/3	4	3	Yes	2	16
2	Right	4	3	1–5	1	>1	>2/3	6	3	Yes	2	15
3	Left	4	3	1–5	1	>1	>2/3	6	1.75	Yes	2	13.75
4	Right	4	3	≥51	4	<1/3	<1/3	2	0	Yes	2	11
5	Right	2	1	0	0	>1	>2/3	6	2	Yes	2	11
6	Left	0	0	21–50	3	1/3–1	1/3–2/3	4	1.5	No	0	8.5
7	Left	0	0	6–20	2	1/3–1	1/3–2/3	4	0	Yes	2	8
8	Left	3	2	1–5	1	<1/3	<1/3	2	0.5	No	0	5.5
9	Right	1	1	0	0	<1/3	<1/3	2	1	Yes	2	6
10	Right	1	1	0	0	<1/3	<1/3	2	1	No	0	4
11	Right	0	0	1–5	1	<1/3	<1/3	2	1	No	0	4
12	Left	0	0	1–5	1	<1/3	ND	1	0.25	No	0	2.25
13	Left	0	0	0	0	0	0	0	0.25	No	0	0.25
14	Right	0	0	0	0	0	0	0	0	No	0	0
15	Left	0	0	0	0	0	0	0	0	No	0	0
16	Right	0	0	0	0	0	0	0	0	No	0	0
17	Left	0	0	0	0	0	0	0	0	No	0	0
18	Left	0	0	0	0	0	0	0	0	No	0	0

Abbreviations: DA, disc area of involvement; FA, foveal area of involvement; ND, not defined.

^a^ Defined on the basis of the no. of retinal quadrants affected, where 1 = ≤2 quadrants affected, 2 = 3 quadrants affected, and 3 = 4 quadrants affected.

^b^ Defined on the basis of the no. of hemorrhages, where 1 = 1–5 hemorrhages, 2 = 6–20 hemorrhages, 3 = 21–50 hemorrhages, and 4 = ≥51 hemorrhages.

^c^ Defined as the sum of macular and foveal whitening scores, where 1 = <1/3 DA or FA, 2 = 1/3–1 DA or 1/3–2/3 FA, and 3 = >1 DA or >2/3 FA.

^d^ Defined on the basis of the presence of papilledema, where 2 = present.

### Percentage of Retinal Vessels With Parasite Sequestration

Five children had causes of death other than CM, had no MR and had sequestration of pRBCs in <10% of their retinal capillaries and in <20% retinal venules (mean [±SD], 5% ± 3% and 9% ± 8%, respectively; Supplementary Table 2). These findings were consistent with the paucity of sequestration seen in the brains of the same patients (Supplementary Table 1). By contrast, all 13 children with histologically proven CM had MR and exhibited pRBC sequestration in a mean (±SD) of 69% ± 21% and 81% ± 16% of retinal capillaries and venules, respectively (*P* < .001; data not shown).

**Figure 2.**
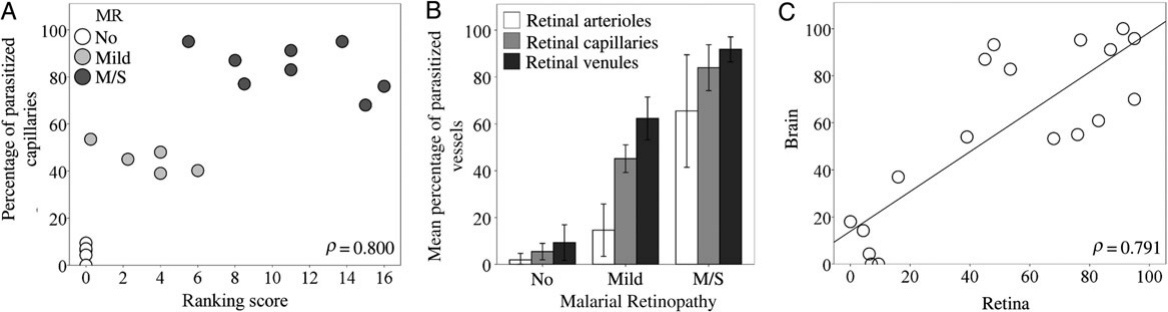
Quantitative assessment of parasitized red blood cell (pRBC) sequestration in retinal and cerebral microvasculature. *A*, Correlation between the overall score for clinical fundoscopy (Table 1) and the percentage of parasitized capillaries (n = 18; *P* < .001). No malarial retinopathy (MR); (No; n = 5) open circles; mild MR (n = 5), light gray circles; moderate/severe (m/s) MR (n = 8), dark gray circles. *B*, Bar chart showing percentages of arterioles, capillaries, and venules affected by pRBC sequestration across the 3 MR classification groups. Results are shown as means ± SDs for each individual MR group. *P* ≤ .001 for all tests across groups per type of vessel, except *P* = .8 for the comparison of means of arterioles in patients with no MR vs those with mild MR. Data were normally distributed (Shapiro-Wilk test). Means were compared using analysis of variance (with the Bonferroni post-hoc test). *C*, Correlation between the percentage of retinal and cerebral capillaries parasitized in the same patients as in panels *A* and *B* (n = 18; *P* < .001).

**Figure 3.**
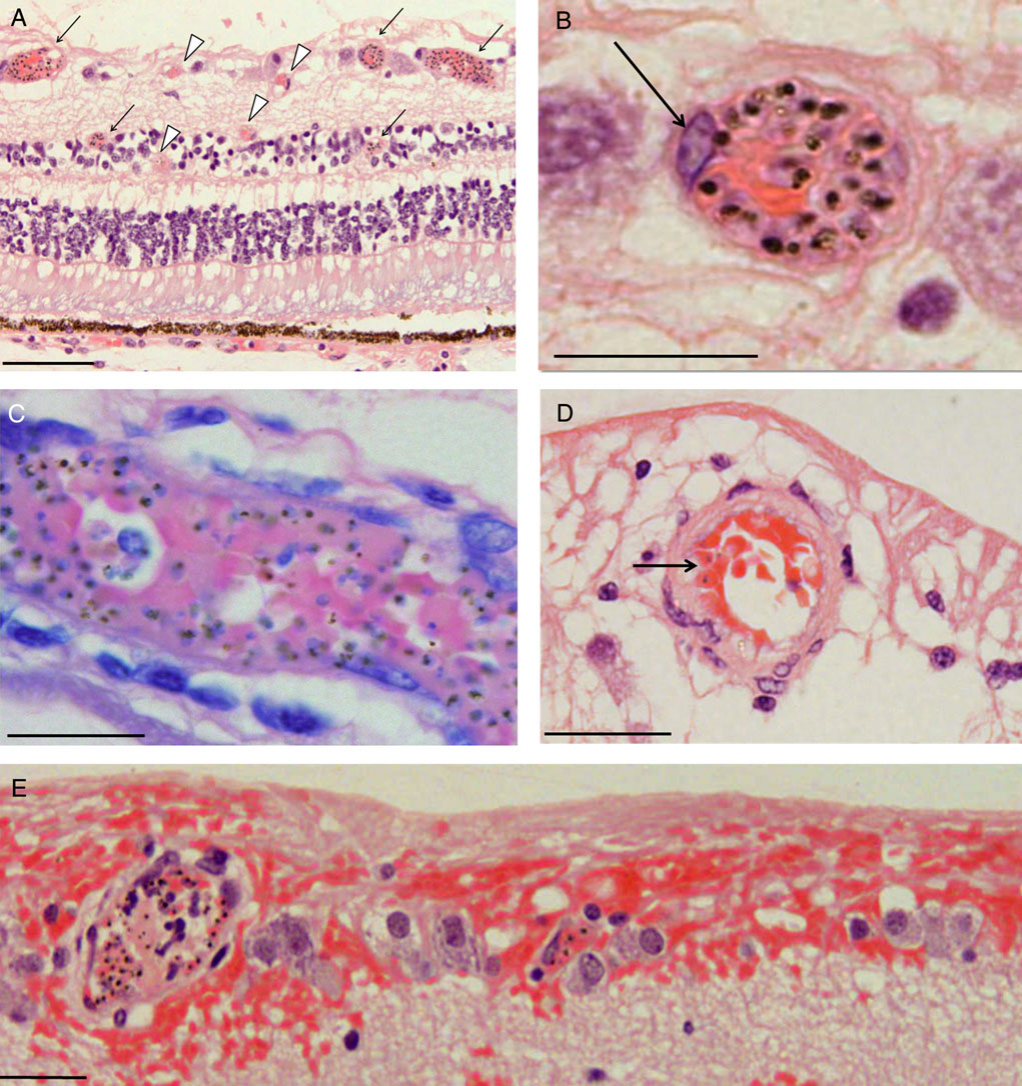
Photomicrographs of parasitized retinal capillaries and venules. *A*, Representative image of retina cross-sections from a moderate/severe MR specimen (patient 3) containing pigmented (mature-form) parasitized red blood cells (pRBCs). Cross-section showing capillaries and venules affected by parasite sequestration are shown (arrows, intense sequestration; arrowheads, low or no sequestration). *B*, High-power image of 1 representative venule with numerous pRBCs. A prominent endothelial cell nucleus is shown (arrow). *C*, A Giemsa-stained venule illustrating pigmented (mature-form) pRBCs (patient 7). *D*, Parasitized arteriole in a cross-section stained by hematoxylin-eosin, with a lower number of pRBCs (arrow; patient 8). The vessel wall is characterized by a thick, pink-stained muscle layer. *E*, Highly parasitized venules with adjacent superficial retinal hemorrhage (patient 4). Section was stained by hematoxylin-eosin. Nonparasitized RBCs stain red, while pigmented pRBCs contain hemozoin, which appears as dark brown spots. Scale bars: 100 μm (*A*); 20 μm (*B*–*D*), and 50 μm (*E*).

The percentage of vessels with pRBC sequestration in the retina measured after death was correlated positively with the grade of MR severity observed before death. This was true for all retinal vessel types (ρ = 0.800 for capillaries [Figure 2*A*]; ρ = 0.823 for arterioles and ρ = 0.851 for venules [data not shown]; *P* < .001 for all). Cases were distributed in 3 clusters, corresponding to the severity of MR. Cases with moderate/severe MR had the highest percentage of parasitized capillaries (mean [±SD], 84% ± 10%; Figure 2*B* and Figure 3*A*–*C*), compared with mild MR (mean [±SD] 45% ± 6%; Supplementary Figure 1). Arterioles were generally less affected than other microvessels in patients with no MR or mild MR (mean [±SD], 2% ± 2% and 15% ± 11% parasitized arterioles, respectively), except in patients with moderate/severe MR, in which the sequestration levels were higher (mean [±SD], 66% ± 24% parasitized arterioles; Figure 2*B* and 3*D* and Supplementary Table 2). Intense sequestration in venules was sometimes associated with retinal hemorrhages (Figure 3*E*).

### Sequestration in Tissues With Neuroectodermal and Nonneuroectodermal Origins

**Figure 4.**
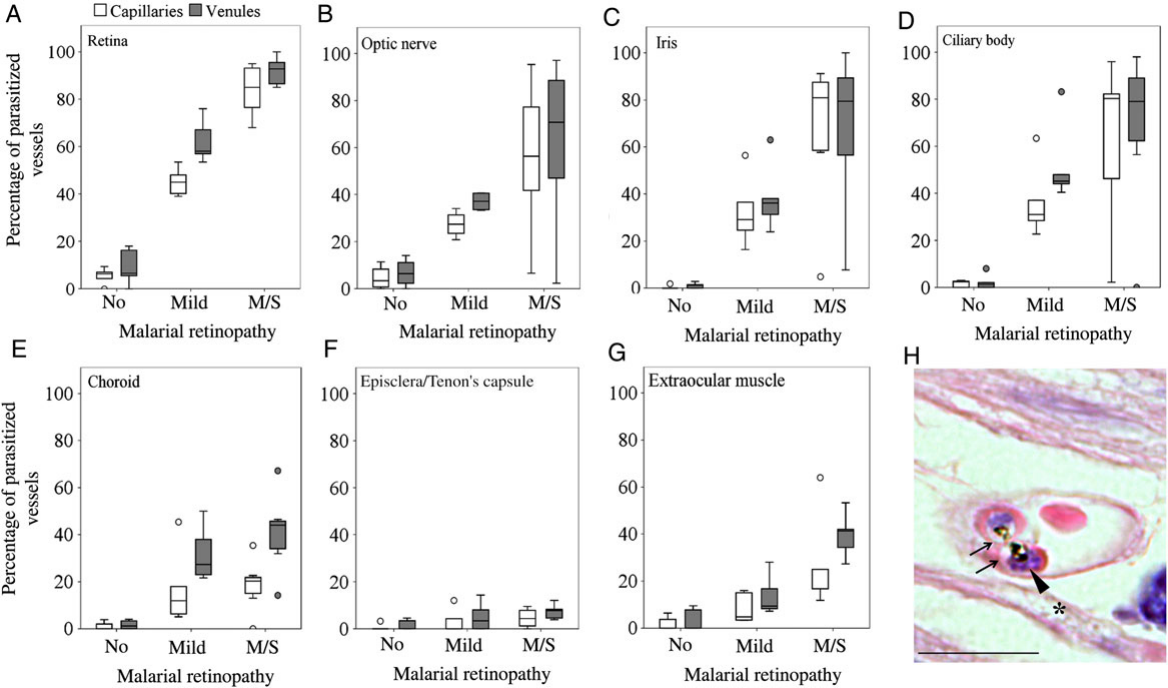
Differential parasitized red blood cell (pRBC) sequestration in ocular microvasculature. *A*–*G*, Box plots showing the percentage of parasitized capillaries (white boxes) and venules (gray boxes) in the different ocular tissues. *A* and *C–G*, There were 5 specimens in the group with no malaria retinopathy (MR), 5 in the mild MR group, and 8 in the moderate/severe (m/s) MR group. *B*, There were 4 specimens in the group with no MR, 4 in the group with mild MR, and 8 in the group with m/s MR n = 8. Data were compared using analysis of variance (with the Bonferroni post-hoc test). *P < .05 and **P < .01, when comparing no MR or mild MR vs m/s MR per vessel type. *P* < .01 for all tests comparing no MR vs mild MR per vessel type, except for choroidal capillaries and episcleral and extraocular muscle vessels. Boxes denote medians and 25th and 75th percentiles. Outliers are reported as white or grey dots outside the boxes. *H*, Representative image of a cross-section of a parasitized capillary from a ciliary body (patient 4), stained by hematoxylin-eosin. Two pigmented mature pRBCs, probably schizonts, are shown. The parasitophorous vacuoles of the parasites inside the RBCs are clearly visible (arrows), with dark brown dots of hemozoin and blue nuclei of new growing parasites (arrowhead). Ciliary muscle fiber is indicated by an asterisk. Scale bar: 20 μm.

To further explore the association between clinically observed MR and pRBC sequestration in neural tissue, we analyzed measurements of sequestration in vessels from the brain and from a range of ocular tissues in the same cohort of patients. We found a positive correlation between the percentages of parasitized capillaries in the retina and brain (Figure 2*C*), and those in the brain were also associated with the MR severity score (ρ = 0.606; *P* < .001; data not shown). The 5 patients without evidence of MR showed sequestration levels of <21% of cerebral capillaries, a threshold above which cerebral pathology is seen and clinical CM is diagnosed [2] (Supplementary Table 1). The distribution of sequestered pRBCs in multiple ocular tissues is shown in Figure 4. The most intense sequestration was seen in tissues containing or closely juxtaposed to a neuroectodermal component (ie, optic nerve head, ciliary body, and iris; Supplementary Materials), similar to that observed in the retina (Figure 4*A*–*D*). Choroidal vessels showed significantly less pRBC sequestration than the retina (Figure 4*E*). Tissues with a nonneuroectodermal origin, such as the episclera/Tenon capsule and extraocular muscles, had the lowest amount of pRBCs of the groups. A positive correlation was found between the percentage of parasitized vessels in ocular vasculature and MR severity grade (both capillaries and venules; *P* < .05; data not shown), except for choroidal capillaries and episcleral capillaries and venules. The maturation stage of sequestered pRBCs in the optic nerve, iris, and ciliary body was the same as that found in the retina (a representative image of pigmented pRBCs is shown in Figure 4*H*).

### Vascular Congestion in Parasitized Retinal Vessels

**Figure 5.**
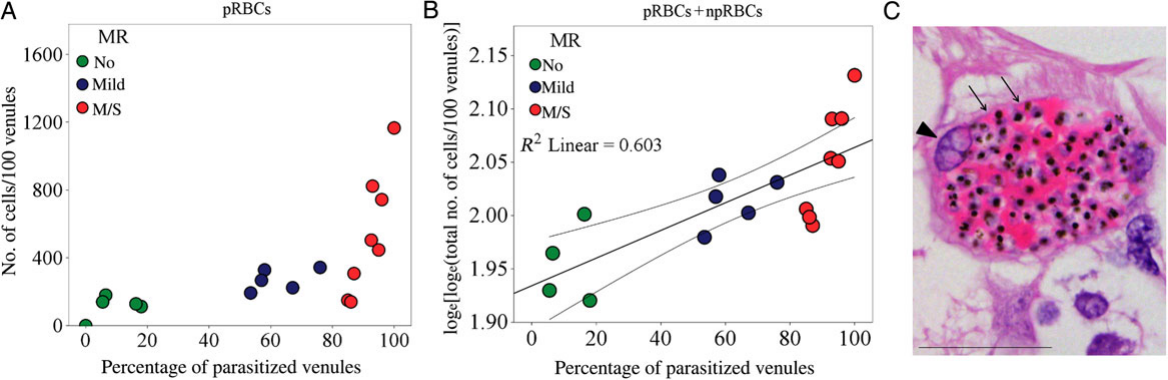
Congestion in retinal venules affected by parasite sequestration. *A*, Scatterplots and ranking correlation between the percentage of parasitized venules and number of parasitized red blood cells (pRBCs) sequestered (n = 18). *B*, Correlation between the number of pRBCs plus nonparasitized RBCs and the percentage of parasitized venules (n = 17; *P* = .001). Linear regression was performed after natural logarithm log_e_-(log_e_) transformation of dependent variables. The coefficient was 1.911. The fit line, R^2^, and 95% confidence interval for the mean are reported on the plot. Patient 18 was removed from the analysis because values for the dependent and independent variables were 0. To evaluate the quality of the model, residual diagnostics were accomplished, and confounders were excluded. *C*, Highly congested venule from a moderate/severe malarial retinopathy (MR) case (patient 3). The section was stained by hematoxylin-eosin. Vessel wall (thin and attenuated) and endothelium nucleus are indicated by arrows and an arrowhead, respectively. Scale bar: 50 μm.

The intensity (calculated as the number of sequestered pRBCs/100 venules) and level (reported as the percentage of microvessels with pRBCs) of sequestration were positively correlated with each other in the retina (ρ = 0.829; *P* < .001; Figure 5*A*). Microvascular congestion, measured as the total number of RBCs in the retinal vasculature (pRBCs plus npRBCs), assessed per 100 parasitized venules, was increased in patients with MR, compared with those without MR (mean [±SD], 843 ± 337 and 352 ± 222, respectively; *P* = .004; data not shown) and positively associated with sequestration levels (ρ = 0.841; *P* < .001; data not shown). We compared means across the 3 MR severity classification groups and found significantly greater congestion in individuals with moderate/severe MR, compared with those without MR (*P* = .008).

A descriptive model using linear regression analysis confirmed the relationship between vascular congestion and levels of sequestration (Figure 5*B*). The number of npRBCs was not increased in vessels without parasite sequestration (ρ = − 0.401; *P* = .11; data not shown), confirming that vascular congestion is restricted to affected venules and is not attributable to postmortem artifacts. Histologically, the lumen of congested venules in moderate/severe MR cases appeared to be packed with both pRBCs and npRBCs (Figure 5*C*).

### Parasite Stage in Relation to MR and CM Severity

**Figure 6.**
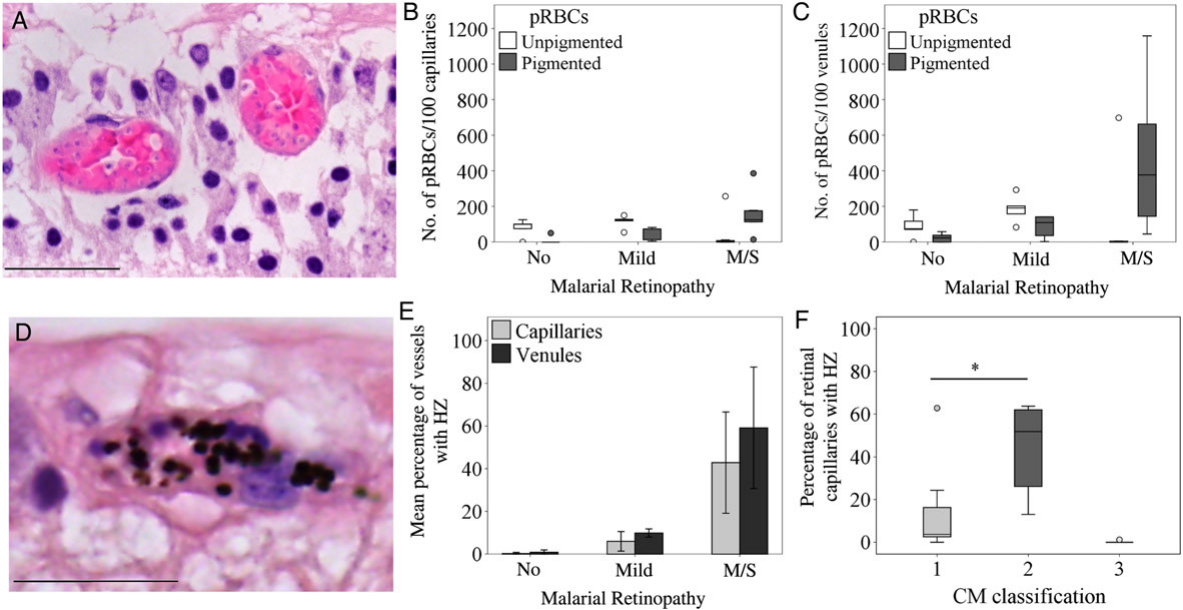
Maturation stage of parasitized red blood cells (pRBCs) in retinal microvasculature. *A*, Representative image of retinal venules containing unpigmented (immature form, with parasite nucleus stained blue inside the infected RBC) stage pRBCs detected in patient 5. *B* and *C*, Box plots showing the relative number of unpigmented (white boxes) and pigmented (gray boxes) pRBCs sequestered in 100 retinal capillaries (*B*) and venules (*C*) across classification groups. There were 5 specimens in the group with no malaria retinopathy (MR), 5 in the mild MR group, and 8 in the moderate/severe (m/s) MR group. *D*, Representative image of retinal microvessel with extraerythrocytic hemozoin (HZ) from patient 2, who had m/s MR (*E*). Bar graphs showing the percentage of retinal capillaries and venules containing extraerythrocytic HZ across classification groups. Results are reported as means ± SD for each individual MR group. *F*, Box plots showing the percentage of parasitized retinal capillaries in the same patients as in panel *E*, but clustered by CM classification. CM1, sequestration of pRBCs in cerebral capillaries; CM2, sequestration of pRBCs in cerebral capillaries plus intravascular and perivascular pathology (Supplementary Table 1); CM3, sequestration of <21% of cerebral capillaries. Boxes denote medians and 25th and 75th percentiles. Data were analyzed by the Kruskal–Wallis test within classification groups. *P ≤ .05 and **P ≤ .01. Scale bars: 50 μm (*A*); 20 μm (*D*).

In moderate/severe MR cases, pigmented pRBCs were the most predominant forms, with unpigmented pRBCs composing <10% of the total amount of pRBCs sequestered, except patient 5 (Figure 6*A*). Unpigmented pRBCs were more commonly observed in retinopathy-negative and mild MR cases (Figure 6*B* and 6*C*). We used hemozoin as an additional marker of parasite maturation across classification groups (Figure 6*D*–*E*). In patients with no MR and those with mild MR, extraerythrocytic hemozoin was seen in <2% of retinal capillaries and <15% of retinal venules. Mean percentages (±SD) of retinal capillaries and venules containing extraerythrocytic hemozoin were high in the moderate/severe MR group (43% ± 24% and 59% ± 28%, respectively). The percentage of retinal capillaries with extraerythrocytic hemozoin was significantly greater in patients with cerebral sequestration plus additional vascular pathology in the brain (CM2) than in patients with cerebral sequestration alone (CM1; *P* < .05; Figure 6*F*), confirming previously published evidence for hemozoin in cerebral capillaries [2].

## DISCUSSION

Clinical observation of MR in children with CM is predictive of cerebral sequestration and death [2]. Our results add important new histological evidence to this finding by showing that the degree of sequestration of pRBCs in the retinal microvasculature discriminates between patients with MR and those without MR, reflecting the severity of sequestration and pathology in the brain. We also provide quantitative evidence for the first time that the severity of MR is correlated with the intensity of sequestration and the parasite maturation stage in retinal microvasculature. Intense sequestration was found in almost all the ocular tissues with neuroectodermal components, and the proportion of retinal capillaries with extraerythrocytic hemozoin predicts CM1 and CM2, 2 histological categories in the brain. The parallels between ocular pathology and brain pathology strongly suggest that features of clinically observed MR closely reflect the CM process, which is taking place throughout the neurovasculature, and that the severity of retinopathy is directly related to the intensity of sequestration.

In all patients without MR, <20% of retinal microvessels contained parasites, which were in smaller numbers and at earlier stages than in MR-positive patients, hypothetically reflecting a nonmalarial coma with incidental parasitemia. These values are comparable with the 21% cutoff of parasitized capillaries in the brain described previously [2], which separated children whose death appeared to be due to CM from those with another cause of death and incidental parasitemia. Among patients with MR, there were significantly different levels (expressed as a percentage of parasitized vessels), as well as intensity (expressed as the number of pRBCs) of parasite sequestration between mild MR and moderate/severe MR.

Parasite sequestration can lead to vessel engorgement [23] and alterations of the vessel wall [24], histologically described as distension of vessels [25] or dilation [19], with impaired venous outflow resulting in vascular congestion. We found an increased number of pRBCs sequestered in retinal venules from retinopathy-positive patients. We have shown histopathological evidence of vascular congestion as a measure of the density of accumulated cells (pRBCs plus npRBCs [19]) and abnormal alterations in retinal venules packed with pRBCs plus npRBCs; a representative image of distended vessel wall and rounded large endothelial nuclei is shown in Figure 5*C*. In a postmortem histopathology study of fatal CM in Vietnamese adults, congestion of heavily parasitized cerebral microvessels was associated with length of coma before death occurred (ie, shorter time to death) [19].

Vascular congestion may be an important part of the retinal disease process leading to MR, possibly reflecting the abnormal accumulation of pRBCs in the cerebral vasculature evolving with CM. Increased vascular congestion may be due to the rosetting process occurring in malaria, by which pRBC can bind to multiple npRBCs, and potentially contributing to microvascular obstruction [26] and CM [27]. When evolving with vessel obstruction, congestion is likely to induce local hypoxia, intravascular thrombosis [22, 28], increased transmural and shear stress, and pathological changes to endothelial cells [23, 29], already described in abnormal vessels observed in children with MR [4, 6].

We are the first to have directly correlated sequestration levels in neurovasculature from the retina and brain, 2 organs of neuroectodermal origin, in children dying of CM. To explore further the association between MR and pRBC sequestration in neural tissues, we analyzed ocular tissues closest to neuroectodermal origin, namely the optic nerve head, ciliary body, and iris (Supplementary Materials), and found that sequestration was as intense as seen in the retina. This similarity is likely due to similar vascular endothelial surface receptor profiles, induced by the tissue in which the vessels are situated and mediating the adhesion of pRBCs within the vessel. The choroid, despite its proximity to the retinal pigment epithelium, which is derived from the neuroectoderm, contained less intense sequestration than other intraocular tissues. Other mechanisms, such as ocular hemodynamics, may play an important role in determining the degree of sequestration in microvessels; the choroid has a high rate of blood flow, roughly 20 times that of the retina [4]. Our observation of greater sequestration in retinal venules and capillaries than in arterioles is consistent with the hypothesis that pRBC sequestration is favored by lower blood velocity, with consequent lower shear stress [4]. These data may reflect how hemodynamics can impact differential sequestration rates in central nervous system, compared with other organs, and confirm the important diagnostic value of MR investigation during life. For this reason, our study may also contribute to defining the mechanism through which the severity of MR predicts cerebral involvement in CM. We have also associated the life-cycle stage of sequestered parasites (seen in the retina after death) with the severity of MR observed during life. This confirms our previous studies on cerebral capillaries in children [2, 16] and evidence from other groups in adults [13], in which unpigmented pRBCs were detected in patients with less evident pathological features (CM1). The presence of more-mature parasites in sequestered pRBCs is associated with more intravascular and perivascular pathology, and this is consistent with previously published evidence of a greater amount of extraerythrocytic hemozoin in cerebral capillaries from CM2 cases [2].

Our analysis is limited by the fact that only 18 patients could be studied, but among these there was a wide spectrum of retinopathy severity, and we identified significant correlations between the grade of MR and the intensities of retinal sequestration in various vessel types. More investigations will be necessary to confirm mechanisms of microvascular pathology associated with sequestration in retina and brain.

In conclusion, we have found evidence that the clinical severity of MR predicts the intensity and level of pRBC sequestration in the neurovasculature of the eyes and brain. Our findings suggest that MR seen in pediatric CM is not an epiphenomenon, but rather a direct reflection of the neurovascular disease process, using the retina as a window to the brain. Retinal examination appears to be a key tool with which to diagnose, follow patients with, and investigate CM during life.

## Supplementary Data

Supplementary materials. are available at *The Journal of Infectious Diseases* online (http://jid.oxfordjournals.org). Supplementary materials consist of data provided by the author that are published to benefit the reader. The posted materials are not copyedited. The contents of all supplementary data are the sole responsibility of the authors. Questions or messages regarding errors should be addressed to the author

Supplementary Data
