# Supplementary material for: Severity of Retinopathy Parallels the Degree of Parasite Sequestration in the Eyes and Brains of Malawian Children With Fatal Cerebral Malaria
Source: J Infect Dis. 2014 Oct 28;211(12):1977–86. doi: 10.1093/infdis/jiu592 (PMC4442623; doi:10.1093/infdis/jiu592)
Supplement: Supplementary Data [file supp_211_12_1977__index.html]

Severity of Retinopathy Parallels the Degree of Parasite Sequestration in Eye and Brain in Malawian Children with Fatal Cerebral Malaria — Severity of Retinopathy Parallels the Degree of Parasite Sequestration in the Eyes and Brains of Malawian Children With Fatal Cerebral Malaria — Severity of Retinopathy Parallels the Degree of Parasite Sequestration in the Eyes and Brains of Malawian Children With Fatal Cerebral Malaria — Supplementary Data 

# Severity of Retinopathy Parallels the Degree of Parasite Sequestration in the Eyes and Brains of Malawian Children With Fatal Cerebral Malaria

## Supplementary Data

Supplementary Data

**Files in this Data Supplement:**

- Supplementary Data - Docx file
- Supplementary Figure 1 - tif file
- Supplementary Table 1 - docx file
- Supplementary Table 2 - docx file
