# Supplementary material for: Severity of Retinopathy Parallels the Degree of Parasite Sequestration in the Eyes and Brains of Malawian Children With Fatal Cerebral Malaria
Source: J Infect Dis. 2014 Oct 28;211(12):1977–86. doi: 10.1093/infdis/jiu592 (PMC4442623; doi:10.1093/infdis/jiu592)
Supplement: Supplementary Data [file supp_jiu592_jiu592supp.docx]

Barrera V et al.

**Severity of Retinopathy Parallels the Degree of Parasite Sequestration in Eye and Brain in Malawian Children with Fatal Cerebral Malaria**

SupplementaRY Material

**Postmortem eye examination and microscopy**

Eyes enucleated during autopsy were opened either horizontally in the pupil-optic nerve plane and the superior and inferior calottes removed, or by an equatorial incision so that the anterior segment was analyzed separately. The eyes were examined macroscopically in 70% v/v ethanol, to confirm the presence of visible pathological features such as retinal orange vessels and hemorrhages. A dissecting microscope (Olympus SZ61 6.7x-45x, Hamburg, Germany), equipped with a high intensity illuminator (Dolan-Jenner Industries Inc, Boxborough, MA, USA) was used, and photographs were taken with a Nikon DS-U1 digital camera (Nikon, Melville, NY, USA). A record of visible pathological features, such as retinal orange vessels and hemorrhages, was taken. Then ocular specimens were dehydrated in ascending concentrations of alcohol, followed by xylene, and embedded in paraffin wax. After wax embedding, tissue blocks were cut in 3-4µm thick sections with a manual rotary microtome (RM2235 from Leica Microsystems Ltd, Milton Keynes, United Kingdom). Slides were from Dako (Dako Ltd UK), staining solutions and Pertex mounting medium from Surgipath (Peterborough, UK). Microscopic investigations were carried out with an Olympus BX60 system microscope equipped with a wide range of Olympus objectives. Images were taken with Olympus DP71 microscope digital camera and Cell imaging software (Olympus, Tokyo, Japan). Total magnifications were: 100, 200, 400 and 1000X. Numerical apertures of the objective lenses were: 0.30, 0.50, 0.75 and 1.30.

**Parasite count in microvessels**

The degree of malaria parasite sequestration was assessed by counting unpigmented and pigmented pRBCs (indicating different malaria parasitic stages) and by-product HZ in individual retinal and cerebral vessel subtypes. HZ identified in the vessel lumen outside RBCs was defined as extraerythrocytic HZ [1]. Capillaries were identified in cross-sections as circular or oval blood vessels, with a diameter between 5-8 µm and with at most one endothelial cell nucleus in the vessel wall [2]. Post-capillary and collecting venules were identified as blood vessels larger in size than a capillary, with 8-50 µm range diameter [3], and distinguished from arterioles by the absence of smooth muscle cells in the vessel wall. The presence of other pathology, such as hemorrhages and thrombosis, was also recorded.

**Differential parasite sequestration in brain and other ocular tissues**

Presence of sequestered pRBCs was previously assessed in capillaries in all areas of the brain [1, 2], and data from cerebral cortex (gray matter) were reported in Figure 2C (for more details see Data Analysis below). Ocular tissues different from the retina were studied in the same standardised fashion described for retinal microvessels. The optic nerve head is the intra-ocular portion of the optic nerve and it is continuous with the neural retina. The choroid lies beneath the retina (or, more specifically, the monolayer of cells known as the retinal pigment epithelium under the neural retina) and is characterized by a complex network of vessels which supplies the nutritional requirement of the adjacent retina, in which the capillaries occupy a layer immediately beneath the retina (choriocapillares). The ciliary body and iris are anterior extensions of the choroid that, all together, comprise the uveal tract. Extra-ocular muscles are skeletal striated muscles responsible for eye movements. Episclera/Tenon’s are extra-ocular supportive connective tissues that surround the globe and extra-ocular muscles and are vascularized by small vessels.

The optic nerve, like retina and brain, is of neuroectodermal origin. The choroid, ciliary body and iris are of mixed embryological derivation. The stroma of these structures has both mesodermal and neural crest elements, while the adjacent epithelia (the retinal pigment epithelium and the epithelium of the ciliary body and iris respectively) are all derived from neuroectoderm [4]. However, inner retinal and choroidal blood flow are significantly different. Data were reported in studies on macaques: ~18ml/100mg/min and ~450ml /100mg/min respectively (assuming specific gravity of blood = 1.06 and macaque retinal weight of 128mg) [5]. Finally, the extraocular muscles and adjacent connective tissues, including episclera and Tenon’s capsule, are thought to be largely derived from mesoderm.

**Data analysis**

Analysis used data from one eye of each subject and from the clinical examination performed within 24 hours before death. Evaluation of stained ocular sections was undertaken by one of the authors (VB). Each case was then scored by at least a second independent observer (one of the authors, PH or one of two other scorers: SB or TW, see acknowledgments), each of whom counted one third of the cases. Brain data were extracted from existing archive [1, 2], in order to compare retinal and cerebral sequestration in capillaries the same cases. Of the 18, data were available for 15 cases; the remaining 3 were analyzed by one of the author (VB), and a second independent scorer (DG, see acknowledgments) in the same standardised fashion described for retinal microvessels. An inter-observer error count of less than 10% was considered acceptable.

**Supplementary Figure Legend**

**Supplementary Figure 1. Parasite sequestration and retinal features in mild MR case n.9.** (A) Color fundus montage taken from patient 9 with mild MR. Arrowheads show retinal whitening limited to temporal macula and 1 quadrant of periphery. (B) Image of retina cross section from case 9, characterized by low-parasitized vessels with few pRBCs (arrows). Scale bar: 100 μm (B).

**References**

1. Milner DA, Valim C, Carr RA, et al. A histological method for quantifying Plasmodium falciparum in the brain in fatal paediatric cerebral malaria. Malar J **2013**; 12:191.

2. Taylor TE, Fu WJ, Carr RA, et al. Differentiating the pathologies of cerebral malaria by postmortem parasite counts. Nat Med **2004**; 10:143-5.

3. Junqueira LC, Carneiro J, Kelley RO. The circulatory system. In Basic Histology. Junqueira LC, Carneiro J, Kelley RO editors. Appleton & Lange, Norwalk, CT. FL. **1995**; 212-217

4. Moe MC, Kolberg RS, Sandberg C, et al. A comparison of epithelial and neural properties in progenitor cells derived from the adult human ciliary body and brain. Experimental eye research **2009**; 88:30-8.

5. Alm A, Bill A. Ocular and optic nerve blood flow at normal and increased intraocular pressures in monkeys (Macaca irus): a study with radioactively labelled microspheres including flow determinations in brain and some other tissues. Experimental eye research **1973**; 15:15-29.

6. White VA, Lewallen S, Beare N, Kayira K, Carr RA, Taylor TE. Correlation of retinal haemorrhages with brain haemorrhages in children dying of cerebral malaria in Malawi. Trans R Soc Trop Med Hyg **2001**; 95:618-21.

7. Dorovini-Zis K, Schmidt K, Huynh H, et al. The neuropathology of fatal cerebral malaria in malawian children. Am J Pathol **2011**; 178:2146-58.
