# Supplementary figures and images for: Severity of Retinopathy Parallels the Degree of Parasite Sequestration in the Eyes and Brains of Malawian Children With Fatal Cerebral Malaria
Source: J Infect Dis. 2014 Oct 28;211(12):1977–86. doi: 10.1093/infdis/jiu592 (PMC4442623; doi:10.1093/infdis/jiu592)

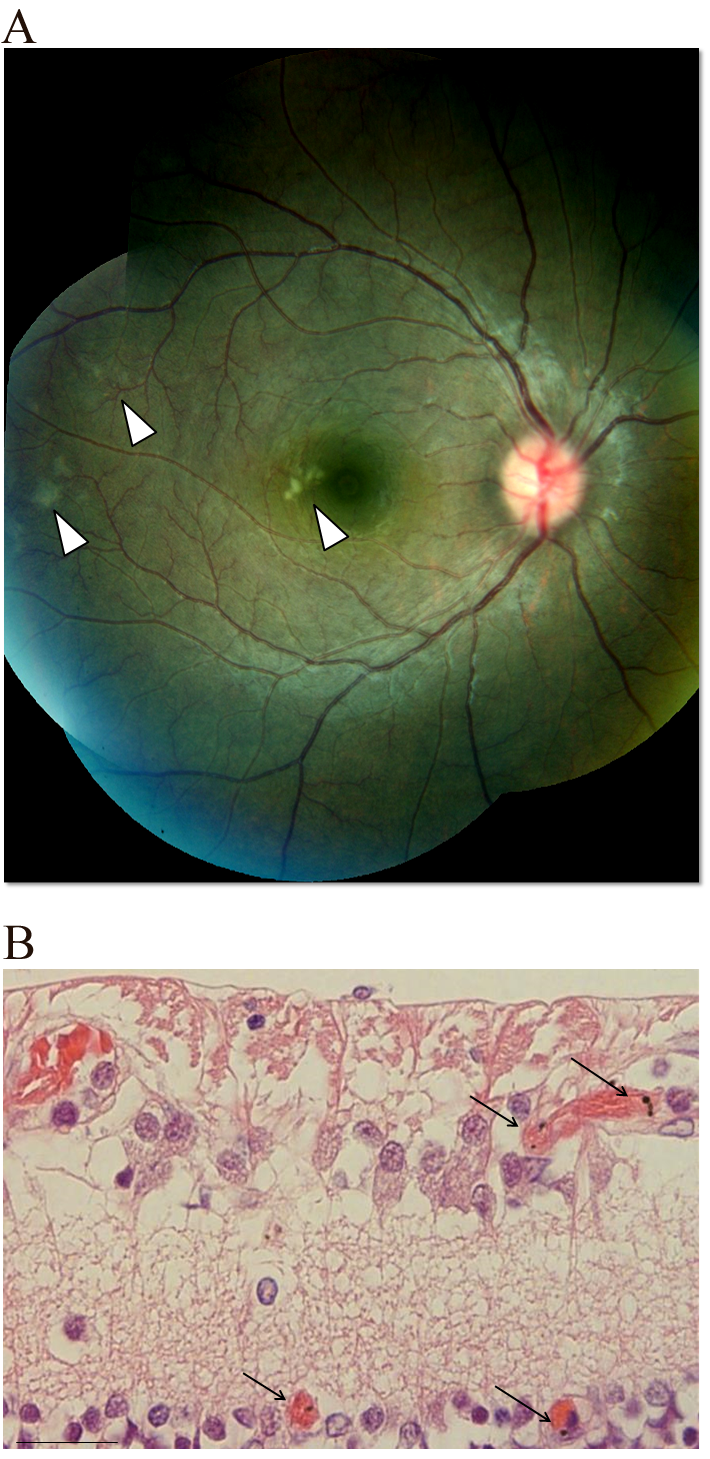

Supplement: Supplementary Data [file supp_jiu592_jiu592supp_fig1.tif]
