# Supplementary material for: Severity of Retinopathy Parallels the Degree of Parasite Sequestration in the Eyes and Brains of Malawian Children With Fatal Cerebral Malaria
Source: J Infect Dis. 2014 Oct 28;211(12):1977–86. doi: 10.1093/infdis/jiu592 (PMC4442623; doi:10.1093/infdis/jiu592)
Supplement: Supplementary Data [file supp_jiu592_jiu592supp_table1.docx]

**Table 2. Clinical records for study participants**.

| **Case n.** | **Autopsy n. ^a^** | **Age (months)** | **MR ^b^** | **CM ^c^** | **HIV** | **Clinical diagnosis** |
| --- | --- | --- | --- | --- | --- | --- |
| 1 | 101 | 25 | m/s | 2 | - | Clinical CM, Pneumonia |
| 2 | 60 | 25 | m/s | 2 | - | Clinical CM |
| 3 | 97 | 72 | m/s | 1 | + | Clinical CM |
| 4 | 99 | 89 | m/s | 1 | + | Clinical CM |
| 5 | 79 | 79 | m/s | 1 | + | Clinical CM |
| 6 | 32 | 18 | m/s | 2 | - | Clinical CM, Severe Malaria Anemia |
| 7 | 98 | 96 | m/s | 2 | + | Clinical CM, Severe Malaria Anemia |
| 8 | 102 | 42 | m/s | 2 | - | Clinical CM |
| 9 | 100 | 41 | mild | 1 | + | Clinical CM |
| 10 | 37 | 06 | mild | 1 | + | Clinical CM, Severe Malaria Anemia |
| 11 | 27 | 20 | mild | 2 | - | Clinical CM |
| 12 | 16 | 51 | mild | 1 | - | Clinical CM |
| 13 | 38 | 84 | mild | 1 | - | Clinical CM |
| 14 | 73 | 96 | no | 3 | - | Non-malarial coma, Pneumonia |
| 15 | 87 | 34 | no | 3 | - | Non-malarial coma, Pneumonia with spread to meninges |
| 16 | 58 | 8 | no | 3 | - | Non-malarial coma, Severe Malaria Anemia, Hepatitis |
| 17 | 31 | 39 | no | 3 | + | Non-malarial coma, Pneumonia, Reye’s |
| 18 | 103 | 21 | no | 3 | - | Non-malarial coma, Pneumonia |

^a^ Autopsy (or PM) number from the autopsy study run in Blantyre, between 1996 and 2010, on the histopathological correlates of severe malaria [2, 9, 19].^b^ MR=malarial retinopathy; m/s=moderate to severe. Last peripheral parasitemia (expressed as asexual pRBCs/μl blood, geometric means reported) was: 21,757 (m/s), 43,212 (mild) and 31,904 (no MR).^c^ CM=cerebral malaria classification system, previously published [2, 18]. The classification included three types: sequestration of pRBCs alone (CM1); sequestration of pRBCs with intra- and perivascular pathology such as hemorrhages, intravascular and perivascular white cells containing HZ, and thrombi (CM2); and sequestration below 21% (CM3). As previously shown [2], the amount of extraerythrocytic HZ in cerebral capillaries was greater in CM2 cases.
