# Supplementary material for: Severity of Retinopathy Parallels the Degree of Parasite Sequestration in the Eyes and Brains of Malawian Children With Fatal Cerebral Malaria
Source: J Infect Dis. 2014 Oct 28;211(12):1977–86. doi: 10.1093/infdis/jiu592 (PMC4442623; doi:10.1093/infdis/jiu592)
Supplement: Supplementary Data [file supp_jiu592_jiu592supp_table2.docx]

**Table S2. Master table of histological counts in retina**

| **Case n.** | **MR** | **CM** | **%Capillaries Parasitized** | **%Capillaries with HZ** | **%Arterioles Parasitized** | **%Arterioles with HZ** | **%Venules Parasitized** | **%Venules with HZ** |
| --- | --- | --- | --- | --- | --- | --- | --- | --- |
| 1 | **m/s** | 2 | 76 | 64 | 25 | 0 | 87 | 76 |
| 2 | **m/s** | 2 | 68 | 60 | 44 | 6 | 85 | 78 |
| 3 | **m/s** | 1 | 95 | 24 | 94 | 10 | 100 | 72 |
| 4 | **m/s** | 1 | 83 | 63 | 65 | 26 | 95 | 82 |
| 5 | **m/s** | 1 | 91 | 0 | 89 | 0 | 96 | 0 |
| 6 | **m/s** | 2 | 77 | 62 | 69 | 3 | 86 | 75 |
| 7 | **m/s** | 2 | 87 | 26 | 86 | 4 | 93 | 35 |
| 8 | **m/s** | 2 | 95 | 44 | 53 | 6 | 93 | 55 |
| **Mean (±SD)** | **m/s** |  | **84 (±10)** | **43 (±24)** | **66 (±24)** | **5 (0-26)*** | **92 (±5)** | **59 (±28)** |
| 9 | **mild** | 1 | 40 | 4 | 16 | 0 | 67 | 8 |
| 10 | **mild** | 1 | 39 | 8 | 6 | 0 | 54 | 10 |
| 11 | **mild** | 2 | 48 | 13 | 7 | 10 | 57 | 12 |
| 12 | **mild** | 1 | 45 | 2 | 33 | 4 | 58 | 8 |
| 13 | **mild** | 1 | 54 | 3 | 10 | 0 | 76 | 12 |
| **Mean (±SD)** | **mild** |  | **45 (±6)** | **6 (±5)** | **15 (±11)** | **0 (0-10)*** | **62 (±9)** | **10 (±2)** |
| 14 | **no** | 3 | 9 | 1 | 0 | 0 | 16 | 0 |
| 15 | **no** | 3 | 6 | 0 | 3 | 0 | 18 | 2 |
| 16 | **no** | 3 | 7 | 0 | 6 | 0 | 6 | 0 |
| 17 | **no** | 3 | 4 | 0 | 0 | 0 | 7 | 0 |
| 18 | **no** | 3 | 0 | 0 | 0 | 0 | 0 | 0 |
| **Mean (±SD)** | **no** |  | **5 (± 3)** | **0 (0-1)*** | **0 (0-6)*** | **0** | **9 (±8)** | **0 (0-2)*** |

Malarial Retinopathy (MR): no=none, m/s = moderate/severe. Cerebral Malaria (CM): see Supplementary Table 1 footnotes. %(Vessel)HZ= percentage of (vessels) with extraerythrocytic hemozoin. SD: standard deviation. Decimals are not shown. * Median (min-max) replaced mean (±SD).
